# ∆^4^-3-oxo-5β-reductase deficiency: favorable outcome in 16 patients treated with cholic acid

**DOI:** 10.1186/s13023-023-02984-z

**Published:** 2023-12-07

**Authors:** Antoine Gardin, Mathias Ruiz, Jan Beime, Mara Cananzi, Margarete Rathert, Barbara Rohmer, Enke Grabhorn, Marion Almes, Veena Logarajah, Luis Peña-Quintana, Thomas Casswall, Amaria Darmellah-Remil, Ana Reyes-Domínguez, Emna Barkaoui, Loreto Hierro, Carolina Baquero-Montoya, Ulrich Baumann, Björn Fischler, Emmanuel Gonzales, Anne Davit-Spraul, Sophie Laplanche, Emmanuel Jacquemin

**Affiliations:** 1 Pediatric Hepatology and Pediatric Liver Transplantation Unit, National Reference Centre for Biliary Atresia and Genetic Cholestasis, FSMR Filfoie, ERN RARE LIVER, Hôpital Bicêtre, Assistance Publique-Hôpitaux de Paris, Faculty of Medicine Paris–Saclay, Le Kremlin-Bicêtre; INSERM UMR-S1193, Hepatinov, University Paris-Saclay, Orsay, France; 2 Pediatric Hepatology, Gastroenterology and Nutrition Unit, Reference Centre for Biliary Atresia and Genetic Cholestasis, Hospices Civils de Lyon - Hôpital Femme Mère Enfant, Bron, France; 3 Pediatric Hepatology and Gastroenterology Unit, University Hamburg-Eppendorf, Hamburg, Germany; 4 Unit of Pediatric Gastroenterology, Digestive Endoscopy, Hepatology and Care of the Child with Liver Transplantation, Dpt. For Women’s and Children’s Health, University Hospital of Padova, Padua, Italy; 5 Pediatric Hepatology, Gastroenterology and Nutrition Unit, MHH Hannover/University Magdeburg, Hannover, Germany; 6 Department of Paediatric Gastroenterology, Hepatology and Nutrition, KK Women’s and Children’s Hospital, Singapore, Singapore; 7 Paediatric Gastroenterology, Hepatology and Nutrition Unit. Complejo Hospitalario Universitario Insular Materno-Infantil, University of Las Palmas de Gran Canaria, Las Palmas, Spain; 8 Pediatric Gastroenterology, Hepatology and Nutrition Unit, Astrid Lindgren’s Children’s Hospital, CLINTEC, Karolinska Institutet, Karolinska University, Stockholm, Sweden; 9 Department of Pediatrics, Tunis Children Hospital, Tunis, Tunisia; 10 Pediatric Hepatology Unit, University Hospital La Paz, Madrid, Spain; 11 Pediatric Genetics, Pablo Tobon Uribe Hospital, Medellin, Colombia; 12 Biochemistry Department, Hôpital Bicêtre, Assistance Publique-Hôpitaux de Paris, Le Kremlin-Bicêtre, France; 13 Biology Unit, Groupe Hospitalier Paris - Saint Joseph, Paris, France

**Keywords:** Bile acid, Genetic cholestasis, *AKR1D1*

## Abstract

**Background:**

Oral cholic acid therapy is an effective therapy in children with primary bile acid synthesis deficiencies. Most reported patients with this treatment have 3β-hydroxy-Δ^5^-C_27_-steroid oxidoreductase deficiency. The aim of the study was the evaluation of cholic acid therapy in a cohort of patients with the rarer Δ^4^-3-oxosteroid 5β-reductase (Δ^4^-3-oxo-R) deficiency.

**Methods:**

Sixteen patients with Δ^4^-3-oxo-R deficiency confirmed by *AKR1D1* gene sequencing who received oral cholic acid were retrospectively analyzed.

**Results:**

First symptoms were reported early in life (median 2 months of age), with 14 and 3 patients having cholestatic jaundice and severe bleeding respectively. Fifteen patients received ursodeoxycholic acid before diagnosis, with partial improvement in 8 patients. Four patients had liver failure at the time of cholic acid initiation. All 16 patients received cholic acid from a median age of 8.1 months (range 3.1–159) and serum liver tests normalized in all within 6–12 months of treatment. After a median cholic acid therapy of 4.5 years (range 1.1–24), all patients were alive with their native liver. Median daily cholic acid dose at last follow-up was 8.3 mg/kg of body weight. All patients, but one, had normal physical examination and all had normal serum liver tests. Fibrosis, evaluated using liver biopsy (n = 4) or liver elastography (n = 9), had stabilized or improved. Cholic acid therapy enabled a 12-fold decrease of 3-oxo-∆^4^ derivatives in urine. Patients had normal growth and quality of life. The treatment was well tolerated without serious adverse events and signs of hepatotoxicity.

**Conclusions:**

Oral cholic acid therapy is a safe and effective treatment for patients with Δ^4^-3-oxo-R deficiency.

**Supplementary Information:**

The online version contains supplementary material available at 10.1186/s13023-023-02984-z.

## Background

Primary bile acid synthesis deficiencies (BASD) are rare inherited autosomal recessive disorders affecting one of the enzymes of the biosynthetic pathway of primary bile acids (BA), in human cholic acid (CA) and chenodeoxycholic acid (CDCA) [1, 2]. The most frequent BASD is the deficiency in 3β-∆^5^-hydroxy-C_27_-steroid oxidoreductase (3β-HSD, OMIM 607765) due to biallelic variants in *HSD3B7*, whereas fewer patients have been reported with a deficiency in ∆^4^-3-oxosteroid-5β-reductase (∆^4^-3-oxo-R, OMIM 235555) due to biallelic variants in *AKR1D1* [2–8]*.* In patients with BASD, the synthesis of primary BA is interrupted or dramatically decreased, resulting in an impairment of bile formation and hepatotoxic/cholestatic atypical BA intermediates are produced upstream of the enzymatic blockade [2, 3, 5, 9]. Most patients are diagnosed in the neonatal period with cholestasis and fat-soluble vitamin deficiencies and the disease progresses towards early cirrhosis and liver failure if left untreated [2–4, 6, 10, 11]. Low serum γ-glutamyltransferase (γGT) level, absence of pruritus and low serum BA level suggest BASD [2, 10]. Diagnosis can be confirmed by the analysis of urinary BA by mass spectrometry, showing absence or abnormally low level of primary BA and accumulation of specific BA intermediates, and/or by identification of pathogenic variants in either *HSD3B7* or *AKR1D1* [2–7, 10]. Oral BA replacement therapy by CA or CDCA is effective in patients with either 3β-HSD or ∆^4^-3-oxo-R deficiencies, with normalization of clinical features and serum liver tests as well as improvement of histology features [2, 11–15]. Oral BA therapy restores the pool of primary BA and bile flow, and downregulates the endogenous production of hepatotoxic BA intermediates [2]. Unlike CDCA [16], CA is not hepatotoxic and long-term CA therapy has been shown to be efficient and safe in patients with BASD [11–15]. Nonetheless, only few patients with ∆^4^-3-oxo-R deficiency treated with CA have been reported [10–13, 17–19], whereas most reported patients have received oral CDCA [6, 15, 17, 20–25], including the largest cohort to date [15]. In this study, we describe the initial presentation and the response to oral CA therapy of 16 patients with ∆^4^-3-oxo-R deficiency with a median treatment duration of 4.5 years.

## Methods

We retrospectively included all patients with ∆^4^-3-oxo-R deficiency diagnosed between 1997 and 2021 who received oral CA replacement therapy with Orphacol^®^ (laboratoire CTRS, Boulogne-Billancourt, France) which is the only drug having a marketing authorization in this indication in Europe. Daily doses of CA range from 5 to 15 mg/kg of body weight (max 500 mg/d). Sixteen patients from 13 families were included in 8 countries (France, n = 7; Germany, n = 3; Italy n = 1; Spain, n = 1; Sweden, n = 1; Tunisia, n = 1; Singapore, n = 1; Colombia, n = 1). Two patients from France (patients A1 and A2) were previously reported with a follow-up ending in 2017 at time of last publication [11, 12]. No patients received CDCA, and 15 out of 16 patients received oral ursodeoxycholic acid (UDCA) therapy at a daily dose of 600 mg/m^2^ of body surface before CA initiation. One patient (A2) is still receiving UDCA therapy along with CA at last follow-up. Clinical features, laboratory measurements and imaging data at initial evaluation (before UDCA initiation), at the time of CA initiation and at last follow-up were retrospectively analyzed. Liver failure was defined as prothrombin time (PT) < 70% of control value after vitamin K supplementation. Liver fibrosis was evaluated on liver biopsy if available using a Metavir score (ranging from F0: no fibrosis to F4: cirrhosis) and/or using liver elastography such as transient elastography (TE) or supersonic shear imaging (SSI) as previously reported in patients with 3β-HSD deficiency [12]. BA analyses in urine samples were mainly performed using liquid chromatography coupled to mass spectrometry and specific 3-oxo-∆^4^ derivatives (7α-hydroxy-3-oxo-Δ^4^-cholenoic acid and 7α, 12α-dihydroxy-3-oxo-Δ^4^-cholenoic acid) were determined in urine and expressed as a percentage of total urinary bile acids (physiological BA and 3-oxo-∆^4^ derivatives) or quantified in µmol/mmol of creatinine when possible. The study was conducted in accordance with guidelines of the Declaration of Helsinki and in compliance with French regulatory authorities for data handling and processing (Registration Number: 20230213184901). In addition, the study was approved by the independent ethics committee of the French-speaking Group for Pediatric Hepatology Gastroenterology and Nutrition (GFHGNP) (Registration Number 2023-44). No patients/families expressed opposition to the use of their data in this retrospective study. Quantitative variables are expressed as median and inter-quartile range Q1–Q3 (IQR) or range (for sample size below 10) and qualitative variables as frequencies. The GraphPad Prism (Dotmatics, Boston, USA) software was used for statistical analysis using two-tailed Mann–Whitney test and a *p* value < 0.05 was considered statistically significant.

## Results

### Initial clinical presentation and response to UDCA in patients with ∆^4^-3-oxo-R deficiency

**Fig. 1:**
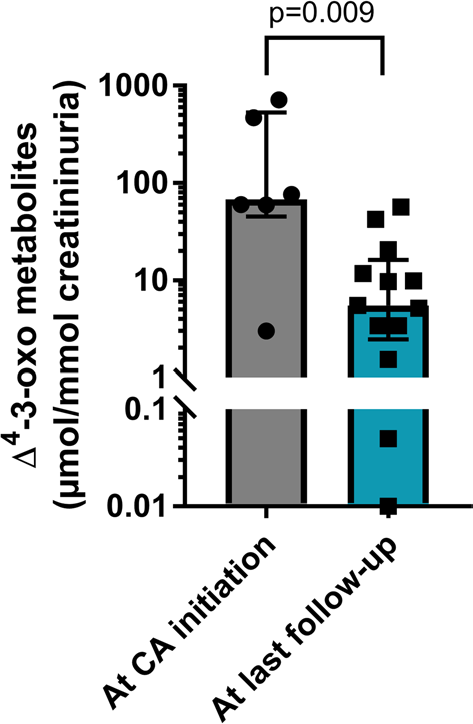
Evolution of 3-oxo-∆^4^ derivatives in urine of patients before (n = 6) and after (n = 13) cholic acid treatment. Data are presented as median ± interquartile range, with a logarithmic scale. Of note, among the 13 patients with quantification of 3-oxo-∆^4^ derivatives at last follow-up, six also had quantification at baseline. Statistical analysis was performed using non-parametric two-tailed Mann–Whitney test. *CA* = *cholic acid*

Over the study period, 16 patients (11 male) with ∆^4^-3-oxo-R deficiency from 13 families were included (Table 1). Consanguinity was reported in two families (15%). One patient was diagnosed in absence of any symptom due to familial screening (Patient D2), whereas all other patients had symptoms from infancy (median 2 months of age, IQR 1.0–3.5, range 1–5) (Table 2). Jaundice was present in 14 out of 16 patients (87%), with pale stools in 6 patients (38%). Three patients (19%) presented with bleedings, including 2 with cerebral hemorrhage, due to profound vitamin K-dependent coagulopathy (PT < 10%, with complete correction following vitamin K supplementation). Eight patients (50%) and 2 patients (13%) displayed clinical hepatomegaly and splenomegaly respectively (Table 3). One patient (Patient A2) exhibited ascites at UDCA initiation and two patients presented with liver failure (Patient A2: PT 35% of control value, Factor V 39% of control value; Patient G1: PT 39% of control value). All 14 patients with jaundice had elevation of aspartate aminotransferase (AST, median 355 IU/L, IQR 267–778), alanine aminotransferase (ALT, median 464 IU/L, IQR 356–724) and conjugated bilirubin (median 128 µmol/L, IQR 68–199) levels in serum (Table 4). As expected, serum γGT (median 45 IU/L, IQR 31–48) and BA levels (median 6 µmol/L, range 2–9) were normal (Table 4), as well as plasma cholesterol level (median 4.1 mmol/L, range 3.3–4.8, n = 12). Plasma fat-soluble vitamins levels were low in 7 out 12 patients for vitamin A (median 174 µg/L, IQR 128–262, N < 200), 11 out of 12 for 25-hydroxy-vitamin D (median 8.6 µg/L, IQR 5.4–15.3, N < 20) and 7 out of 10 for vitamin E (median 3.7 mg/L, IQR 1.7–5.1, N < 5). Ten patients underwent a liver biopsy before CA initiation and liver histology showed cholestasis in 8 patients (80%), giant cell transformation of hepatocytes in 8 patients (80%), mild ductular reaction in 7 patients (70%), portal or lobular inflammation in 6 patients (60%) and fibrosis in 7 patients (70%; F1 n = 3; F2 n = 2; F3, n = 1; F4 n = 1) (Table 5). No patient presented with histological signs of biliary obstruction. Six patients had elastography evaluation before CA initiation, with values corresponding to fibrosis levels ranging from F0 to F4 (Table 5). Fourteen patients had urinary BA analysis at diagnosis, with massive increase of 3-oxo-∆^4^ derivatives (median 89% of total urinary BA, IQR 68–94%), quantified in 6 patients (median 68 µmol/mmol of creatinine, range 3–717) (Fig. 1). All patients had biallelic pathogenic variants in the *AKR1D1* gene (Table 1 and Additional file 1: Table 1), corresponding to classes 4 or 5 of the American College of Medical Genetics (ACMG) classification [26]. Most of them were missense variants (22 out of 26 alleles), some of them being recurrent such as p.Arg261Cys/His (4 families), p.Asp81Val (3 families), p.Arg266Gln (2 families) and p.Arg307Cys (2 families). All patients except the one diagnosed through familial screening received oral UDCA therapy for a median duration of 2.8 months (IQR 2.0–6.2, range 1–153) before receiving CA. Jaundice disappeared in only 6 out of 14 patients with UDCA treatment (Table 3). Serum liver tests improved in 8 patients out of 14 with UDCA treatment and normalized in only 5 patients (Table 4). Nonetheless, in the two patients with liver failure at UDCA initiation serum liver tests did not improve and two further patients developed liver failure while being treated with UDCA (Patient F1: PT 56% of control value; Patient M1: 59% of control value).

**Table 1:** Demographics and *AKR1D1* variants in 16 children with ∆^4^-3-oxo-R deficiency

Patient	Sex	Ethnicity	*AKR1D1* gene analysis^a^	
			Allele 1	Allele 2
A1	F	Caucasian	c.398C > G (p.Pro133Arg)	c.781C > T (p.Arg261Cys)
A2	F	Caucasian	c.398C > G (p.Pro133Arg)	c.781C > T (p.Arg261Cys)
B1	M	Caucasian & South Asian	c.593C > T (p.Pro198Leu)	c.797G > A (p.Arg266Gln)
C1	M	Chinese	c.614delT (p.Leu205Profs*2)	c.919C > T (p.Arg307Cys)
D1	F	Caucasian	c.539T > C (p.Leu180Pro)	c.856dupA (Ile286Asnfs*3)
D2	M	Caucasian	c.539T > C (p.Leu180Pro)	c.856dupA (Ile286Asnfs*3)
E1	M	Arab	c.242A > T (p.Asp81Val)	c.242A > T (p.Asp81Val)
F1	M	Caucasian & Arab	c.242A > T (p.Asp81Val)	c.782G > A (p.Arg261His)
F2	M	Caucasian & Arab	c.242A > T (p.Asp81Val)	c.782G > A (p.Arg261His)
G1	M	Caucasian	c.856-2A > C	c.782G > A (p.Arg261His)
H1	F	Caucasian	c.782G > A (p.Arg261His)	c.793C > A (p.Gln265Lys)
I1	M	Chinese	c.580-13T > A	c.919C > T (p.Arg307Cys)
J1	M	Caucasian	c.662C > T (p.Pro221Leu)	c.662C > T (p.Pro221Leu)
K1	M	Arab	c.242A > T (p.Asp81Val)	c.242A > T (p.Asp81Val)
L1	M	South Asian	c.797G > A (p.Arg266Gln)	c.797G > A (p.Arg266Gln)
M1	F	Hispanic	c.332T > C (p.Leu111Pro)	c.332T > C (p.Leu111Pro)

^a^AKR1D1 reference in NCBI = NM_005989

**Table 2:** First symptoms and cholic acid therapy data in 16 children with ∆^4^-3-oxo-R deficiency

Patient	Age at first symptoms (months)	First symptoms	Age at CA initiation (months)	Age at last FU (years)	Daily dose of CA at last FU	
					mg/kg	mg
A1	1	Jaundice	9	25.0	7.9	450
A2	1	Jaundice	9	25.0	8.3	450^a^
B1	4.8	Jaundice Cerebral hemorrhage	7.4	3.6	9.4	150
C1	2	Jaundice	5.3	10.0	9.0	250
D1	3	Cerebral hemorrhage	4.5	2.6	12.0	150
D2		None (family screening)	68	6.7	4.0	100
E1	1	Jaundice	159	16.9	4.1	400
F1	2	Jaundice	6	5.3	7.4	200
F2	2	Jaundice	31	5.3	7.9	200
G1	1	Jaundice	3.1	5.4	14.2	350
H1	2	Jaundice	5	4.9	10.0	150
I1	4	Jaundice, bleeding	7	5.0	8.3	135
J1	1	Jaundice	38	13.8	4.3	250
K1	2.5	Jaundice	25	4.4	7.3	150
L1	4	Jaundice	5	4.3	13.1	200
M1	5	Jaundice	9	6.6	8.4	250
Median	2		8.1	5.4	8.3	200

^a^Patient A2 is also receiving oral UDCA at 200 mg/d at last follow-up along with CA. *CA* cholic acid,* FU* follow-up

**Table 3:** Features of liver disease before and after cholic acid therapy in 16 children with ∆^4^-3-oxo-R

Patient	Clinical features			Findings on abdominal US	
	At UDCA initiation	At CA initiation	At last follow-up	At CA initiation	At last follow-up
A1	Jaundice, HMG	Jaundice, HSM	Normal	HMG	Normal
A2	Jaundice, HSM ascites	Jaundice, HSM ascites	Normal	HSM ascites	Normal
B1	Jaundice, HMG	HMG	Normal	Normal	Normal
C1	Jaundice, HMG	HMG	Normal	Normal	Normal
D1	Normal	Normal	Normal	Normal	Normal
D2	NA^a^	Normal	Normal	Normal	Normal
E1	Jaundice, HMG	HSM	HSM	HSM	HSM
F1	Jaundice	Jaundice	Normal	SMG	Normal
F2	Jaundice	Normal	Normal	–	Normal
G1	Jaundice	Jaundice	Normal	Normal	Normal
H1	Jaundice, HMG	HMG	Normal	Normal	Normal
I1	Jaundice	Jaundice	Normal	Normal	Normal
J1	Jaundice, HMG	HMG	Normal	HMG	Normal
K1	Jaundice, HSM	Jaundice, HMG	Normal	–	Normal
L1	Jaundice	Jaundice, SMG	Normal	SMG	Normal
M1	Jaundice	Jaundice	Normal	Normal	Normal

^a^Patient D2 did not receive oral UDCA therapy. – indicates missing data. *CA* cholic acid, *HMG* hepatomegaly, *HSM* hepatosplenomegaly, *NA* not applicable, *SMG* splenomegaly, *UDCA* ursodeoxycholic acid, *US* ultrasonography

**Table 4:** Serum liver tests before and after cholic acid therapy in 16 children with ∆^4^-3-oxo-R deficiency

Patient	At UDCA initiation				At CA initiation				At last follow-up			
	ALT (IU/L)	γGT (IU/L)	Total/direct bilirubin (µmol/L)	Bile acid (µmol/L)	ALT (IU/L)	γGT (IU/L)	Total/direct bilirubin (µmol/L)	Bile acid (µmol/L)	ALT (IU/L)	γGT (IU/L)	Total bilirubin (µmol/L)	Bile acid^b^ (µmol/L)
A1	1540	48	313/217	7	284	138	51/41	39	11	13	4	5
A2	1110	48	314/224	2	144	48	304/206	130	35	30	7	12
B1	840	32	186/159	9	146	33	15	42	13	15	4	4
C1	391	53	57/53	6	45	13	4	30	10	8	4	11
D1	47	9	19/–	6	13	9	7/3	27	24	17	7	1
D2^a^	NA	NA	NA	NA	22	9	4	1	16	13	6	1
E1	656	31	231/204	–	26	16	5	26	18	21	10	–
F1	468	45	149/125	–	1563	54	289/201	180	33	8	3	9
F2	218	45	121/99	–	25	7	4	6	14	10	0	–
G1	791	48	247/212	–	997	106	259/225	56	29	16	5	11
H1	389	33	56/44	8	42	9	5/2	–	15	10	8	–
I1	346	17	96/57	5	582	18	68/43	–	16	12	6	–
J1	464	93	196/184	–	280	140	43/34	–	13	13	8	3
K1	366	56	87/78	–	26	16	7	–	21	20	4	5
L1	225	29	78/65	–	328	37	83/61	5	19	11	4	19
M1	627	31	176/130	–	1509	48	380/296	–	18	13	5	–
N	< 50	< 50	< 17/ < 4	< 10	< 50	< 50	< 17/ < 4	< 10	< 50	< 50	< 17/ < 4	< 10

^a^Patient D2 did not receive oral UDCA therapy

^b^Not performed in fasting. – indicates missing value. *ALT* alanine aminotransferase, *CA* cholic acid, *γGT* γ-glutamyltransferase, *N* normal range, *NA* not applicable, *UDCA* ursodeoxycholic acid

**Table 5:** Liver fibrosis evaluation before and after cholic acid CA therapy in children with Δ^4^-3-oxo-R deficiency

Patients	At CA initiation			At last follow-up		
	Liver biopsy^a^	Elastography^b^		Liver biopsy^a^ (duration of CA therapy)	Elastography^b^ (duration of CA therapy)	
A1	F3	–		F1/F2 (17y)	SSI (24y):	5.5 kPa (F0)
A2	F4	–		F3 (17y)	SSI (24y):	7.6 kPa (F1)
B1	–	SSI:	12.5 kPa (F4)	–	SSI (3y):	6.2 kPa (F0)
C1	F1	–		–	SSI (9y):	5.5 kPa (F0)
D1	F0	TE:	7.0 kPa (F1)	–	TE (2y):	3.2 kPa (F0)
D2	F0	TE:	3.1 kPa (F0)	–	TE (1y):	3.6 kPa (F0)
E1	F2	TE:	5.6 kPa (F0)	–	TE (3.5y):	4.7 kPa (F0)
F1	F1	SSI:	5.5 kPa (F0)	–	SSI (5y):	5.7 kPa (F0)
F2	–	SSI:	3.1 kPa (F0)	–	SSI (3y):	4.5 kPa (F0)
G1*	F0	–		–		–
J1	F1	–		F1 (14y)		–
M1	F2	–		F1 (5.5y)		–

^a^Metavir score stages fibrosis from F0 (absence of fibrosis) to F4 (cirrhosis)

^b^The methods of analysis (TE vs. SSI) are indicated and equivalence for fibrosis Metavir score is provided, based on [12]. *No evaluation of fibrosis was performed in the follow-up, but this patient displayed normal clinical examination and normal serum liver tests at last follow-up. – indicates missing value. *CA* cholic acid, *SSI* shear stress imaging, *TE* transient elastography

### Cholic acid therapy and long-term follow-up

All patients eventually received oral CA therapy at a median age of 8.1 months (IQR 5.3–26.9, range 3.1–159) once the diagnosis was confirmed (Table 2). Of note, one patient (Patient E1) was initially diagnosed as undetermined cholestasis and treated with oral UDCA for 13 years until a diagnosis of ∆^4^-3-oxo-R deficiency was made. The initial median dose of oral CA was 7.4 mg/kg/d (IQR 5.8–9.6). Within 6–12 months of CA therapy, jaundice and liver biochemistry normalized in all patients (Tables 3 and 4) and liver failure resolved in patients A2, F1, G1 and M1. The dose of CA was adapted to weight gain during growth and to urinary BA analysis in order to maintain low 3-oxo-∆^4^ derivatives, but was usually not increased above 8–10 mg/kg/d as long as serum liver tests remained normal. At last follow-up, patients received a median dose of 8.3 mg/kg/d (IQR 7.4–9.5) and one patient (patient A2) also received concomitant UDCA (200 mg/d) (Table 2). After a median CA replacement therapy of 4.5 years (IQR 2.9–6.8, range 1.1–24), all patients were alive with their native liver. All patients had normal physical examination and abdominal ultrasonography, except patient E1 who still exhibited hepatosplenomegaly (Table 3). Of note, this patient was diagnosed and started on CA therapy at 13 years of age. All patients displayed normal AST, ALT, γGT and bilirubin levels in serum (Table 4). Serum alpha-fetoprotein was slightly elevated in the youngest patient (Patient D2, 18 µg/L at 18 months of age (N < 10)) and normal in all other patients tested (n = 10). No patient displayed nodules on liver ultrasonography. Unlike patients with 3β-HSD deficiency [11, 12], no gallstones or kidney cysts were reported in our cohort. Four patients had a liver biopsy in the follow-up and liver histology showed no significant cholestasis or inflammation. Liver fibrosis scored in these biopsies was either stable or improved (F1: n = 2; F1/F2: n = 1; F3, n = 1) compared to previous liver biopsies (Table 5). Liver fibrosis assessed at last follow-up by liver elastography in nine patients showed values corresponding to a Metavir F0 score in 8 patients and F1 in one patient (Table 5). Of note, in patient G1, no evaluation of fibrosis was performed in the follow-up, but this patient displayed normal clinical examination and normal serum liver tests at last follow-up.

No patient had failure to thrive with a median Z-score for age of + 0.3 (IQR − 0.7 to + 1.3, range − 1 to + 4.6, n = 13) and − 0.2 (IQR − 0.5 to + 0.4, range − 0.9 to + 2.0, n = 13) for weight and height at last follow-up respectively. At last follow-up, no patient received vitamin supplementation beyond what is recommended for the general population. Plasma levels of vitamin A or E were within normal ranges in all evaluated patients (n = 13 and 14 respectively), whereas 2 patients out of 14 had a mild 25-hydroxy-vitamin D deficiency. All patients had urinary BA analysis at last follow-up. These analyses showed a significant reduction in 3-oxo-∆^4^ derivatives in all patients that were nonetheless still detectable at low levels in most patients (trace amounts). The metabolites were quantified at last follow-up in 13 patients, including the 6 who had quantification at baseline, with a median value of 5.5 µmol/mmol of creatininuria (IQR 3–12, range 0–56) corresponding to a decrease of 12-fold compared to the initial median level (Fig. 1).

The treatment with oral CA was well tolerated without severe adverse events. One patient (Patient L1) exhibited pruritus with weight loss 12 months after CA therapy initiation in absence of CA overdosing and with normal serum liver tests including serum bile acids. This event spontaneously resolved during follow-up. All patients had a normal quality of life and one patient (patient A2) had an uneventful pregnancy with CA treatment (already reported in (12)).

## Discussion

BASD are severe inherited liver diseases leading to death or liver transplantation in absence of treatment [2]. Oral primary BA therapy can restore the physiological bile flow, prevent fat-soluble vitamin deficiencies and reduce the synthesis of hepatotoxic atypical BA intermediates [2, 9, 11–14]. Thus, oral primary BA can halt the progression of liver disease and even allow regression of liver fibrosis [11–15]. Most of the patients in published cohorts have 3β-HSD deficiency; only few cohorts of patients with ∆^4^-3-oxo-R deficiency treated with oral primary BA have been published [10–15]. The most detailed cohort consists of 12 patients with ∆^4^-3-oxo-R deficiency treated with CDCA [15]. Although most patients had good metabolic control and normal serum liver tests, one patient required liver transplantation and the authors insisted on the careful titration of CDCA required in order to avoid hepatotoxicity [15].

In our study, we report the initial presentation and detailed outcome of 16 patients with ∆^4^-3-oxo-R deficiency treated with oral CA. Similar to CDCA, CA is an agonist of the FXR nuclear receptor. It enables the down-regulation of BA synthesis and therefore reduces the production of atypical BA intermediates [2]. Also, the biliary excretion of CA allows the resumption of bile secretion [2]. In contrast to CA and CDCA, UDCA is only choleretic as it does not have the ability to reduce the production of 3-oxo-∆^4^ derivatives [2]. This likely explains the limited improvement of serum liver tests and clinical features observed in some patients with oral UDCA therapy, as previously reported [6]. In opposite, CA therapy normalized the manifestations of the disease in almost all patients, thus confirming previously published results in patients with 3β-HSD deficiency [11–14] and in small cohorts of patients with ∆^4^-3-oxo-R deficiency [10–13, 17–19]. Although there are reports of unfavorable outcomes (death or liver transplantation) in some patients with ∆^4^-3-oxo-R deficiency treated with oral CA [10, 13] or CDCA [6, 15, 17, 25] in the literature, in our experience, all patients were alive with their native liver at a median follow-up of 4.5 years after CA therapy initiation. All patients but one had normal physical examination and all patients had normal growth and normal serum liver tests. No liver tumors were reported. Liver fibrosis was mostly assessed at last follow-up using non-invasive techniques. Few data are available in the literature regarding the interpretation of liver stiffness values in children with BASD [12]. Nonetheless, liver stiffness values obtained using elastography in our patients during follow-up are within the range associated with little/no fibrosis when using cutoffs developed for adult patients, and similar to the ones observed in patients with 3β-HSD deficiency [12]. Combined with studies of liver biopsy available in some patients, this suggests that liver fibrosis improved in all patients in which it was initially present and that early treatment has the potential to reverse installed fibrosis, as described for patients with 3β-HSD deficiency [11, 12]. It also prevented the development of fibrosis in patients without fibrosis at CA therapy initiation (patients D1 & D2). Metabolic control was good in all patients, with significant decrease of 3-oxo-∆^4^ derivatives in urine with CA treatment. But in contrast to patients with 3β-HSD deficiency treated with a similar dose of oral CA (6–7 mg/kg/d) [11, 12], we could not achieve complete disappearance of these atypical BA intermediates in urine, suggesting that a complete downregulation of their synthesis is harder to achieve compared to that of 3β-HSD deficiency atypical metabolites and/or that their biliary excretion is lower [9, 12]. Oral daily CA dose ranged between 7 and 10 mg/kg of body weight in most patients. However, the presence of residual 3-oxo-∆^4^ derivatives in urine did not prevent a favorable outcome in our cohort. No significant adverse events were reported with CA therapy. In addition, no signs of hepatotoxicity (based on serum liver tests) were observed in patients treated with CA, including in the four patients who presented with decompensated liver disease or liver failure. This safety profile of CA treatment contrasts with the one of CDCA for which signs of hepatotoxicity have been previously reported in patients with BASD [15]. Some of the limitations of our study include its retrospective nature, the rarity of patients with ∆^4^-3-oxo-R deficiency, the lack of a homogenous dose escalation protocol among different centers, as well as the fact that liver biopsy was not performed in all patients during the follow-up. The latter is mainly explained by the favorable outcome observed in all of our patients.

## Conclusion

In conclusion, these data show that, in our experience, oral CA therapy is a safe and effective treatment for patients with ∆^4^-3-oxo-R deficiency. It is expected that, as observed in 3β-HSD deficiency, oral CA therapy will allow affected children to reach adulthood with a quality of life similar to the general population in absence of liver transplantation.

### Supplementary Information

**Additional file 1**. Table S1.

## Data Availability

All data generated or analyzed during this study are included in this published article and its supplementary information files.

## Abbreviations

3β-HSD: 3β-Hydroxy-Δ^5^-C_27_-steroid oxidoreductase
Δ^4^-3-oxo-R: Δ^4^-3-Oxo-steroid-5β-reductase
ALT: Alanine aminotransferase
AST: Aspartate aminotransferase
BA: Bile acids
BASD: Bile acid synthesis deficiency
CA: Cholic acid
CDCA: Chenodeoxycholic acid
γGT: γ-Glutamyltransferase
IU: International units
PT: Prothrombin time
SSI: Supersonic shear imaging
TE: Transient elastography
UDCA: Ursodeoxycholic acid
