# Supplementary material for: ∆4-3-oxo-5β-reductase deficiency: favorable outcome in 16 patients treated with cholic acid
Source: Orphanet J Rare Dis. 2023 Dec 7;18:383. doi: 10.1186/s13023-023-02984-z (PMC10704681; doi:10.1186/s13023-023-02984-z)
Supplement: Supplementary file 1 — Additional file 1. Table S1. [file 13023_2023_2984_MOESM1_ESM.docx]

| **Variant** | **Already reported** | | **Present in GnomAD** | **In silico prediction scores** | | | **ACMG variant classification^1^ (justification)** |
| --- | --- | --- | --- | --- | --- | --- | --- |
|  | **In Clinvar^1^** | **In PubMed** |  | **Polyphen 2** | **SIFT** | **CADD Phred^2^** |  |
| c.242A>T  (p.Asp81Val) | Class 3 | No | 0.0000043 %  Nhomalt : 0 | Probably damaging (0.994) | Deleterious | 29.7 | **Class 4** (PM1, PM2, PM3, PP3, PP4) |
| c.332T>C  (p.Leu111Pro) | Class 4 (2019)  Class 3 (2017) | No | 0.0000080 %  Nhomalt : 0 | Probably damaging (1.0) | Deleterious | 27.1 | **Class 4** (Clinvar) |
| c.398C>G  (p.Pro133Arg) | Class 5  (2010, patients A1 & A2)  Class 3 (2022) | Yes (1)  Reduced AKR1D1 activity (2) | 0.000018 %  Nhomalt : 0 | Probably damaging (0.999) | Deleterious | 25.8 | **Class 5** (Clinvar) |
| c.539T>C  (p.Leu180Pro) | No | No | Absent | Probably damaging (1.0) | Deleterious | 29.6 | **Class 4** (PM2, PM3, PP3, PP4) |
| c.580-13T>A (p.Val194Glnfs*17 /  p.Val194Glyfs*23) | No | Yes (3,4)  Induce abnormal splicing (5) | 0.000020 %  Nhomalt : 0 | NA | NA | 19 | **Class 4** (PM2, PS3) |
| c.593C>T  (p.Pro198Leu) | Class 5 (2003)  Class 3 (2013)  Class 4 (2016) | Yes (6,7) | 0.000028 %  Nhomalt : 0 | Possibly damaging (0.457) | Tolerated | 22.8 | **Class 4** (Clinvar) |
| c.614delT (p.Leu205Profs*2) | No | Yes (7) | Absent | NA | NA | 33 | **Class 5** (PVS1, PM2, PP4) |
| c.662C>T  (p.Pro221Leu) | No | No | 0.000012 %  Nhomalt : 0 | Benign (0.017) | Deleterious | 23.8 | **Class 4** (PM1, PM2, PP3, PP4) |
| c.781C>T  (p.Arg261Cys) | Class 5  (Patients A1 & A2) | Yes (1,8)  Reduced AKR1D1 activity (2) | 0.000014 %  Nhomalt : 0 | Probably damaging (1.0) | Deleterious | 29.3 | **Class 5** (Clinvar) |
| c.782G>A  (p.Arg261His) | Class 3 | No | 0.00011%  Nhomalt : 0 | Probably damaging (1.0) | Deleterious | 29.6 | **Class 4** (PM1, PM2, PM3, PM5, PP4) |
| c.793C>A  (p.Gln265Lys) | No | No | Absent | Probably damaging (0.999) | Deleterious | 25.5 | **Class 4** (PM1, PM2, PP4) |
| c.797G>A  (p.Arg266Gln) | Class 4 | Yes (3,4,7) | 0.000067 %  Nhomalt : 0 | Probably damaging (1.0) | Deleterious | 30 | **Class 4** (Clinvar) |
| c.856-2A>C  (p.(?)) | No | No | Absent | NA | NA | 34 | **Class 5** (PVS1, PM2, PP4) |
| c.856dupA (Ile286Asnfs*3) | No | No | Absent | NA | NA | - | **Class 5** (PVS1, PM2, PP4) |
| c.919C>T  (p.Arg307Cys) | No | Yes (4,9) | 0.0000071 %  Nhomalt : 0 | Probably damaging (1.0) | Deleterious | 24.9 | **Class 4** (PM1,PM2, PM3, PP3, PP4) |

**Additional Table 1. ACMG classification of AKR1D1 variants identified in our cohort**. ^1^Based on the 2015 ACMG guidelines. ^2^CADD: a score greater of equal to 10 indicates that the variants are predicted to be the 10% most deleterious substitutions that you can do to the human genome, and a score greater or equal to 20 indicates the 1% most deleterious.

*ACMG= American College of Medical Genetics; Nhomalt= Number of homozygous alternate variant carriers; NA= not applicable.*

**References:**

1. Gonzales E, Cresteil D, Baussan C, Dabadie A, Gerhardt MF, Jacquemin E. SRD5B1 (AKR1D1) gene analysis in Δ4-3-oxosteroid 5β-reductase deficiency: evidence for primary genetic defect. J Hepatol. 2004;40:716–8.

2. Drury JE, Mindnich R, Penning TM. Characterization of Disease-related 5β-Reductase (AKR1D1) Mutations Reveals Their Potential to Cause Bile Acid Deficiency. J Biol Chem. 2010;285:24529–37.

3. Chen JY, Wu JF, Kimura A, Nittono H, Liou BY, Lee CS, et al. AKR1D1 and CYP7B1 mutations in patients with inborn errors of bile acid metabolism: Possibly underdiagnosed diseases. Pediatr Neonatol. 2020;61:75–83.

4. Zhao J. Primary ∆4-3-oxosteroid 5β-reductase deficiency: Two cases in China. World J Gastroenterol. 2012;18:7113.

5. Zhao J, Qiu YL, Wang L, Li ZD, Xie XB, Lu Y, et al. Recurrent AKR1D1 c.580-13T>A Variant: A Cause of Δ4-3-Oxosteroid-5β-Reductase Deficiency. J Mol Diagn. 2023;25:227–33.

6. Lemonde HA, Custard EJ, Bouquet J, Duran M, Overmars H, Scambler PJ, et al. Mutations in SRD5B1 (AKR1D1), the gene encoding 4-3-oxosteroid 5 -reductase, in hepatitis and liver failure in infancy. Gut. 2003;52:1494–9.

7. Zhang MH, Setchell KD, Zhao J, Gong JY, Lu Y, Wang JS. Δ4-3-oxosteroid-5β-reductase deficiency: Responses to oral bile acid therapy and long-term outcomes. World J Gastroenterol. 2019;25:859–69.

8. Seki Y, Mizuochi T, Kimura A, Takahashi T, Ohtake A, Hayashi SI, et al. Two neonatal cholestasis patients with mutations in the SRD5B1 (AKR1D1) gene: diagnosis and bile acid profiles during chenodeoxycholic acid treatment. J Inherit Metab Dis. 2013;36:565–73.

9. Wang HH, Wen FQ, Dai DL, Wang JS, Zhao J, Setchell KD, et al. Infant cholestasis patient with a novel missense mutation in the AKR1D1 gene successfully treated by early adequate supplementation with chenodeoxycholic acid: A case report and review of the literature. World J Gastroenterol. 2018;24:4086–92.
